# An evaluation of the DEXLIFE ‘self-selected’ lifestyle intervention aimed at improving insulin sensitivity in people at risk of developing type 2 diabetes: study protocol for a randomised controlled trial

**DOI:** 10.1186/s13063-015-1042-1

**Published:** 2015-11-18

**Authors:** Grainne M. O’Donoghue, Aileen Kennedy, Gregers Stig Andersen, Eoin Durkan, Tanja Thybo, Margaret Sinnott, John J. Nolan, Donal J. O’Gorman

**Affiliations:** Centre for Preventive Medicine, School of Health and Human Performance, Dublin City University, Dublin, Ireland; Steno Diabetes Center, Gentofte, Denmark; Vhi Healthcare Ltd, Dublin, Republic of Ireland

**Keywords:** Insulin sensitivity, Lifestyle intervention, Prevention, Randomised controlled trial, Self-selected, Type 2 diabetes

## Abstract

**Background:**

With the global escalation of type 2 diabetes and evidence consistently showing that its onset can be prevented or delayed by changing lifestyle behaviours, there is an urgent need to translate practical, affordable and acceptable interventions from the research setting into the real world. One such approach to lifestyle interventions might be the introduction of a programme in which the individual is provided with choice and facilitated to ‘self-select’ an exercise programme. Previous research has shown that this is likely to be less resource intensive, an essential requirement for success outside the controlled research environment, while at the same time promoting positive responses relating to adherence, competence and self-efficacy, essential attributes for long-term success. Through a two-group parallel-randomised controlled trial, this study aims to assess the clinical and psychological impact of the DEXLIFE ‘self-selected’ lifestyle modification programme in adults at risk of developing type 2 diabetes.

**Methods/design:**

A total of 360 subjects at risk of developing type 2 diabetes are randomly assigned in a 1:3 ratio to a control (*n* = 90) or intervention arm (*n* = 270). Randomization is stratified by age, sex and body mass index. The control arm receives general information on lifestyle and diabetes risk. The intervention group participate in a 12 week ‘self-selected’ supervised exercise training programme accompanied with dietary advice to improve food choices. Participants are given access to Dublin City University Sport (an on-campus gym) and asked to perform four exercise classes per week. Dublin City University Sport offers over 50 classes per week, many of which are medically supervised. If weight loss is indicated, reduction in total calorie intake by 600 kcal/day is advised. Common to all food plans is <10 % saturated fat intake, as well as a dietary fibre intake of >15 g/1000 kcal.

Insulin sensitivity is the primary outcome measure. Secondary outcome measures include glucose function, fitness, body composition, anthropometrics, heart rate variability, lipid profiles, blood pressure, physical activity levels, dietary intake and quality of life.

**Discussion:**

"Self-selected" lifestyle intervention has not previously been evaluated in type 2 diabetes prevention and if shown to be successful could be implemented in practice immediately.

**Trial registration:**

Current Controlled Trials: ISRCTN66987085.

**Electronic supplementary material:**

The online version of this article (doi:10.1186/s13063-015-1042-1) contains supplementary material, which is available to authorized users.

## Background

Patients, physicians, health care organizations and policy planners are struggling with the worldwide rise in the incidence of type 2 diabetes mellitus. Recent decades have seen its prevalence reach epidemic proportions and it is now recognized as the fastest growing chronic disease globally. In 2012, the International Diabetes Federation estimated that more than 370 million people worldwide had diabetes mellitus and that treating it accounted for at least 11 % of total health care expenditures in adults (US $471 billion) [1]. It is anticipated that, unless this exponential increase is slowed or reversed, 592 million people will have diabetes by 2035, with roughly the same number again having prediabetes [1]. Approximately 50 % of these people are undiagnosed and may have diabetes for 5–10 years before clinical identification [1].

It is therefore, not surprising that many of these individuals have diabetes-related complications at the time of diagnosis, with substantially greater risk of mortality and significantly greater treatment costs. Preventing the onset of disease by detecting the risk of progression at an earlier stage and implementing effective prevention programmes is vital in controlling this epidemic. DEXLIFE is an EU FP7 funded project designed to identify biomarkers that better predict the onset of type 2 diabetes in high-risk individuals [2]. A key element to the DEXLIFE project is the implementation of a lifestyle intervention programme that can be used to track changes in biomarkers with risk factors for type 2 diabetes. The design of this intervention had to ensure variation in dietary and physical activity patterns so that identified biomarkers could be applied to everyday life and not just in a controlled research setting.

Large-scale lifestyle modification trials in Europe, the USA and elsewhere have shown that the onset of type 2 diabetes can be prevented or delayed among adults at high risk. The Finnish Diabetes Prevention Study and the US Diabetes Prevention Project revealed a 58 % risk reduction in the progression from impaired glucose tolerance to type 2 diabetes [3–6]. Although great variation in individual responses to dietary modification and exercise training has long been recognized, ‘diet and exercise prescription’ for prevention of diabetes remains largely empirical and much research is required to understand the variation in behavioural and physiological responses to lifestyle modification and to optimally match programmes to individual needs and abilities [7].

Interventions that are delivered through diverse organizations that are embedded in the societal structure and are offered to heterogeneous populations, allowing for individual preferences relating to exercise and diet, are required [8–11]. One such approach to ‘real-world’ lifestyle interventions might involve focusing on the concept of providing choice and facilitating ‘self-selection’ [12, 13]. When self-selection is permitted, the real and perceived rigidity of conventional exercise and dietary guidelines is avoided [14]. It has been shown that by providing choice, an individual’s autonomy is supported and there is an increased perception of control over behaviour [15], resulting in positive adherence, competence and self-efficacy, essential attributes for long-term successful lifestyle change [15, 16].

A systematic review conducted in 2009 [14] examined whether people when ‘left to their own devices’ select exercise intensities that are conducive to cardiorespiratory fitness and health enhancement. A total of 33 studies were included and the overall results showed that self-selected intensity approximated or exceeded the minimum level of the range recommended by the American College of Sports Medicine. Furthermore, from a physiological perspective, self-selected exercise programmes have reported exercise intensities close to the individual’s ventilatory or lactate threshold, resulting in positive physiological improvement [17, 18].

In a similar manner to exercise studies, dietary modification can also improve insulin sensitivity [19, 20] and both are influenced by adherence. Even in individuals who initially achieve a desirable response to dietary restriction, long-term adherence is poor and weight regain can lead to discouragement and, ultimately, discontinuation [21]. Implicit in the concept of adherence is choice and agreed goal setting, treatment planning, and implementation of the regimen. Individuals adhere well when they have been involved in the decision making process, when it makes sense to them and when their preference is taken into account [22]. Highly prescriptive programmes may not be the most successful approach for longer-term health behaviour change; they undermine the individual’s sense of autonomy, generate resistance, do not consider what is important to the individual and do not work in the majority of cases [23]. Facilitating ‘self-selection’ by providing information and guidance may enhance longer-term adherence [22, 23].

To date, there are no large trials investigating the effectiveness of a ‘self-selected’ exercise and dietary lifestyle intervention on individuals at risk of developing type 2 diabetes. The purpose of the DEXLIFE intervention is to determine whether the provision of choice and the facilitation of self-selection is an effective means of reducing modifiable clinical, anthropometric, fitness and psychosocial risk factors associated with the development of type 2 diabetes in high-risk individuals.

## Methods/design

### Study design

The study design is a two-group parallel-randomised controlled trial.

### Setting

The trial is conducted in the Centre for Preventive Medicine, Dublin City University in Dublin, Ireland. The intervention programme is delivered in Dublin City University Sport, an on-campus gym. Dublin City University Sport offers approximately 50 classes per week, varying in intensity and type. This gym is unique in so far as, alongside the usual gym classes such as spinning, aerobics and Pilates (30 classes), it provides a chronic illness rehabilitation programme (MedEx Wellness Programme) that caters for individuals with established cardiovascular disease, diabetes, cancer, heart failure, peripheral vascular disease and pulmonary disorders (20 classes). These programmes are medically supervised and carefully monitored in a casual and friendly atmosphere. Participants in this study could chose to participate in any of the 50 classes on offer [24].

### Hypothesis

The hypothesis tested in this study is that there will be a greater increase in mean change in insulin sensitivity among those participating in the DEXLIFE ‘self-selected’ lifestyle intervention than in those in the control group, who will receive general information on lifestyle and diabetes risk.

### Sample size and power calculation

A 3:1 ratio will be used to randomize a total of 360 participants; 270 to the intervention group and 90 to the control group. We expect a 10 % attrition rate, which will leave 243 and 81 participants in the intervention and control groups, respectively. Based on the effect of lifestyle intervention on insulin sensitivity reported in a previous study [25], we have calculated that, with this sample size and with a power of 0.8, it will be possible to detect a 17 % (equivalent to 0.36 standard deviation) difference in insulin sensitivity between the intervention group and the control group in this study.

### Participants: recruitment and eligibility

Potential participants are identified in three ways. Information sessions are held locally; within the university and in local sports clubs. General practitioners and pharmacists within a 10 km radius of Dublin City University have been contacted and provided with trial information and eligibility criteria and consent forms for potential participants. An online screening tool (FINDRISC) is accessible on the DEXLIFE website. If an individual scores >12, an email is automatically generated to the Dublin City University recruitment team and the potential participant is contacted. Finally, Vhi Healthcare, Ireland’s largest health insurance company, and one of the partners in DEXLIFE, will identify suitable clients from their database of policy holders. Letters will be sent out providing information about the study, together with an invitation to an information session in Dublin City University, where the study is explained in detail by the research team. All potential participants are provided with research study information sheets and consent forms. Once signed consent forms are returned, the Dublin City University research team responds with a phone call, answering any questions relating to the study and scheduling appointments for baseline assessments over the following month.

On the baseline assessments days, the researchers administer a series of clinical, anthropometric, behavioural and psychological tests, measures and surveys, as described in Additional file 1. In addition, sociodemographic data on age, sex, self-reported ethnicity, occupational status, educational attainment and marital status, family history of diabetes or cardiovascular disease and lifestyle (smoking, alcohol consumption, physical activity levels and dietary intake) are collected. Additional file 2 outlines the movement of the participants through the stages of recruitment to the study. Following the baseline health assessments, eligibility for the study is determined on the following criteria.

#### Inclusion criteria

Adults, aged between 18–75 years, who report a sedentary lifestyle (<150 minutes physical activity per week) and show at least one of the following diabetes risk factors are eligible to participate:

1. Impaired fasting glucose (fasting plasma glucose levels >5.6 to <7 mmol)
2. Impaired glucose tolerance (two-hour plasma glucose levels of >7.8 to <11.1 mmol)
3. FINDRISC questionnaire score >12 (1 in 6 chance of developing type 2 diabetes in the next 10 years)

#### Exclusion criteria

At the time of recruitment, individuals who have any of the following are not eligible to participate in the study: (i) type 2 diabetes mellitus; (ii) severe cardiovascular, respiratory or renal disease; (iii) active cancer; (iv) neuromuscular, musculoskeletal or rheumatoid disorders exacerbated by exercise; (v) peak aerobic capacity >50 ml/kg·min; (vi) >5 % decrease in body weight in the previous 3 months or (vii) significant cognitive or mental illness.

### Randomization process

An allocation sequence is obtained using the RALLOC command implemented in the statistical software package STATA/IC 12.1 (StataCorp LP, USA) and used to randomize eligible participants in a 3:1 ratio to intervention (DEXLIFE ‘self-selected’ lifestyle programme) or control. Randomization is stratified by age, sex and body mass index using random blocks of four, to ensure a balance of participant characteristics in each group. Participants will be randomly allocated to either intervention or control by the project manager according to the allocation sequence. Because of the nature of the intervention, neither the participants nor the research team are blinded to allocation.

### Description of intervention and control groups

#### Intervention

The DEXLIFE lifestyle intervention is a 12 week ‘self-selected’ supervised exercise training programme accompanied with dietary advice, with an overall aim of increasing insulin sensitivity. Each participant is given access to Dublin City University Sport (an on-campus gym) and asked to perform four moderately intense exercise sessions (45–60 minutes) per week. Rather than performing individualized programmes, participants are encouraged to join group fitness classes. Dublin City University Sport offers over 50 classes per week, many of which are medically supervised. The majority of classes combine dynamic cardiovascular and resistance exercises. These classes offer a safe environment to all participants, particularly older participants, who may be unfamiliar with a gym environment and nervous about exercising for the first time, and who may require a little more support from both a social and a medical perspective. Participants are free to self-select and attend any of the available classes in Dublin City University Sport that they feel best suit their daily routine, exercise preference and ability. Participants are encouraged to attend the gym for all exercise sessions. When this is not feasible, exercise programmes are performed at home with remote support from the DEXLIFE team in Dublin City University.

A 3 day estimated food diary is used to assess dietary intake. Once completed, the participant meets with a dietician together review the diary, identifying unhealthy food choices and develop a plan to modify these choices. If weight loss is indicated, reduction in total calorie intake by 600 kcal/day is advised. A detailed review of the dietary record is performed with the participant and healthy alternatives or suggestions are provided to replace unhealthy components of the diet. Common to all food plans is <10 % saturated fat intake as well as a dietary fibre intake of >15 g/1000 kcal, irrespective of whether weight loss is a requirement or not.

To optimize adherence, an exercise diary, pedometer and regular follow-up telephone contact are utilized. Individuals are asked to record all exercise sessions in an online diary, providing details of the type, time and intensity of exercise completed. A pedometer is given to all participants, so that they can record daily number of steps and number of steps per specific exercise session if they are completing their exercise sessions at home The exercise diary is closely monitored by the research team and contact is made if the diary is not been completed for more than a couple of days.

#### Control

The control group are given general information about lifestyle and diabetes risk. This is achieved individually (15 minutes), and some printed material is delivered; with the message to reduce weight and increase physical activity levels.

### Outcome measures

The primary outcome measure in the study is the change in insulin sensitivity. Insulin sensitivity will be estimated from the 3 hour oral glucose tolerance test. First, the change in the area under the curve for glucose divided by the change in the area under the curve for insulin will be used. Secondly, the Homeostatic Model for Insulin Resistance [25] will be used. Finally, an estimate of insulin sensitivity will be derived from the index proposed by Matsuda and DeFronzo [26], which is calculated according to the following formula:

$$ \frac{10,000}{\sqrt{\mathrm{fasting}\kern0.5em \mathrm{glucose}\kern0.5em \left[\mathrm{mg}/\mathrm{dl}\right]\times \mathrm{fasting}\kern0.5em \mathrm{insulin}\kern0.5em \left[\mathrm{mU}/\mathrm{ml}\right]\times \mathrm{mean}\kern0.5em \mathrm{glucose}\kern0.5em \left[\mathrm{mg}/\mathrm{dl}\right]\times \mathrm{mean}\kern0.5em \mathrm{insulin}\kern0.5em \left[\mathrm{mU}/\mathrm{ml}\right]}} $$

Secondary outcomes will also be measured at baseline and follow-up namely: 2 hour postprandial blood glucose on a 75 g oral glucose tolerance test, maximal fitness levels, body composition and anthropometric measures, lipid profile, blood pressure, heart rate variability, dietary adherence and free-living physical activity levels. Participants will also complete a number of self-reported surveys relating to quality of life, self-efficacy and barriers and facilitators to exercise participation. Additional file 1 outlines the definitions and measurement techniques relating to all outcome measures employed.

### Analysis plan

At follow-up (13–14 weeks post-baseline assessment) all participants will undergo the same biomedical, anthropometric and psychological measures administered at baseline. At the end of the study, data will be cleaned and checked for normality and transformed as needed. Two-tailed *t* tests, analysis of covariance (ANCOVA) and multiple linear regression models will be applied to compare between-group and within-group changes in means before and after intervention for primary and secondary outcome measures. Analysis and reporting will be in line with the Consolidated Standards of Reporting Trials (CONSORT) guidelines; primary analyses will be conducted on an intention-to-treat basis.

### Ethics

Ethical approval for the study was obtained in July 2012 from the Dublin City University Research Ethics Committee (DCUREC/2012/080). Funding is provided through DEXLIFE, an EU FP7 project (grant agreement no: 279228). Written informed consent will be obtained from each participant. Participants are able to withdraw from the trial at any time. All information disclosed in the study will be kept confidential and participants will not be identifiable in any material published as part of the study in any way. All data are stored without any identifying details. At all stages of research, the data will be stored using a password-protected computer and a secure locked cabinet; and all correspondence between research team members will be conducted using secure email.

## Discussion

This clinical trial is being conducted as part of the DEXLIFE project (www.dexlife.eu). DEXLIFE is an EU funded project whose objective is to identify novel metabolomic and lipodomic biomarkers that predict progression towards type 2 diabetes mellitus and are responsive to lifestyle intervention in people at increased risk [2]. This will be achieved by identifying novel biomarkers in longitudinal cohorts of subjects who have progressed from normal glucose tolerance to diabetes and determining the biomarkers that are correlated with improved glucose tolerance following the 12 week lifestyle intervention described in this protocol.

## Trial status

Recruitment started September 2012 and is expected to be complete by September 2015.

## Additional files

Additional file 1:
**Additional details of outcome measures used in the study.** (DOCX 15 kb) Additional file 2:
**Participant flow through the recruitment process.** (PDF 170 kb)

## Abbreviations

ANCOVA: analysis of covariance
CONSORT: Consolidated Standard of Reporting Trials
