# Supplementary material for: An evaluation of the DEXLIFE ‘self-selected’ lifestyle intervention aimed at improving insulin sensitivity in people at risk of developing type 2 diabetes: study protocol for a randomised controlled trial
Source: Trials. 2015 Nov 18;16:529. doi: 10.1186/s13063-015-1042-1 (PMC4652413; doi:10.1186/s13063-015-1042-1)
Supplement: Additional file 1: — Additional details of outcome measures used in the study. (DOCX 15 kb) [file 13063_2015_1042_MOESM1_ESM.docx]

|  | **Outcome** | **Definition and measurement** |
| --- | --- | --- |
| 1. | Insulin sensitivity | An increase in insulin sensitivity as measured by the Matsuda formula, area under the curve for glucose and insulin and the HOMA model as measured during a 3-hr 75g OGTT |
| 2. | Postprandial glucose dysfunction | Mean reduction in two-hour postprandial (PP) blood glucose on a 75 gram oral glucose tolerance test (OGTT) |
| 3. | Fitness levels | An incremental exercise test using a 12 lead ECG was performed to establish maximal aerobic capacity (VO_2 max_) |
| 4. | Body composition | Dual-energy X-ray absorptiometry (DEXA) measured percentage bone, muscle and fat mass |
| 5. | Anthropometrics | Height, weight, waist circumference, hip circumference, waist hip ratio |
| 6. | Abdominal adiposity | Ultrasound measures of subcutaneous and visceral fat distribution |
| 7. | Serum lipids | Total cholesterol, HDL, LDL and triglycerides |
| 8. | Blood pressure | Systolic and diastolic blood pressure |
| 9. | Heart rate variability | Measures beat to beat interval variations in arterial pulse signals |
| 10. | Dietary intake | 3 day food diary and a short questionnaire that provides detail on core foods regularly eaten analysed using WISP (Weighed Intake Software Program) |
| 11. | Physical activity levels | 7 days recording using the *actigraph* *GT3x* accelerometer and the RPAQ (Regular Physical Activity Questionnaire) self-report questionnaire |
| 12. | Psychological evaluation | Quality of life : The Short Form (36) Health Survey (SF36)  Self-efficacy: Barriers self-efficacy scale (BARSE)  Ideas about exercise: Exercise Benefits and Barriers Scale |

Table 1: Outcomes, definitions and measurements techniques
