# Supplementary figures and images for: An evaluation of the DEXLIFE ‘self-selected’ lifestyle intervention aimed at improving insulin sensitivity in people at risk of developing type 2 diabetes: study protocol for a randomised controlled trial
Source: Trials. 2015 Nov 18;16:529. doi: 10.1186/s13063-015-1042-1 (PMC4652413; doi:10.1186/s13063-015-1042-1)

## RECRUITMENT

via local sources, Vhi  
and website

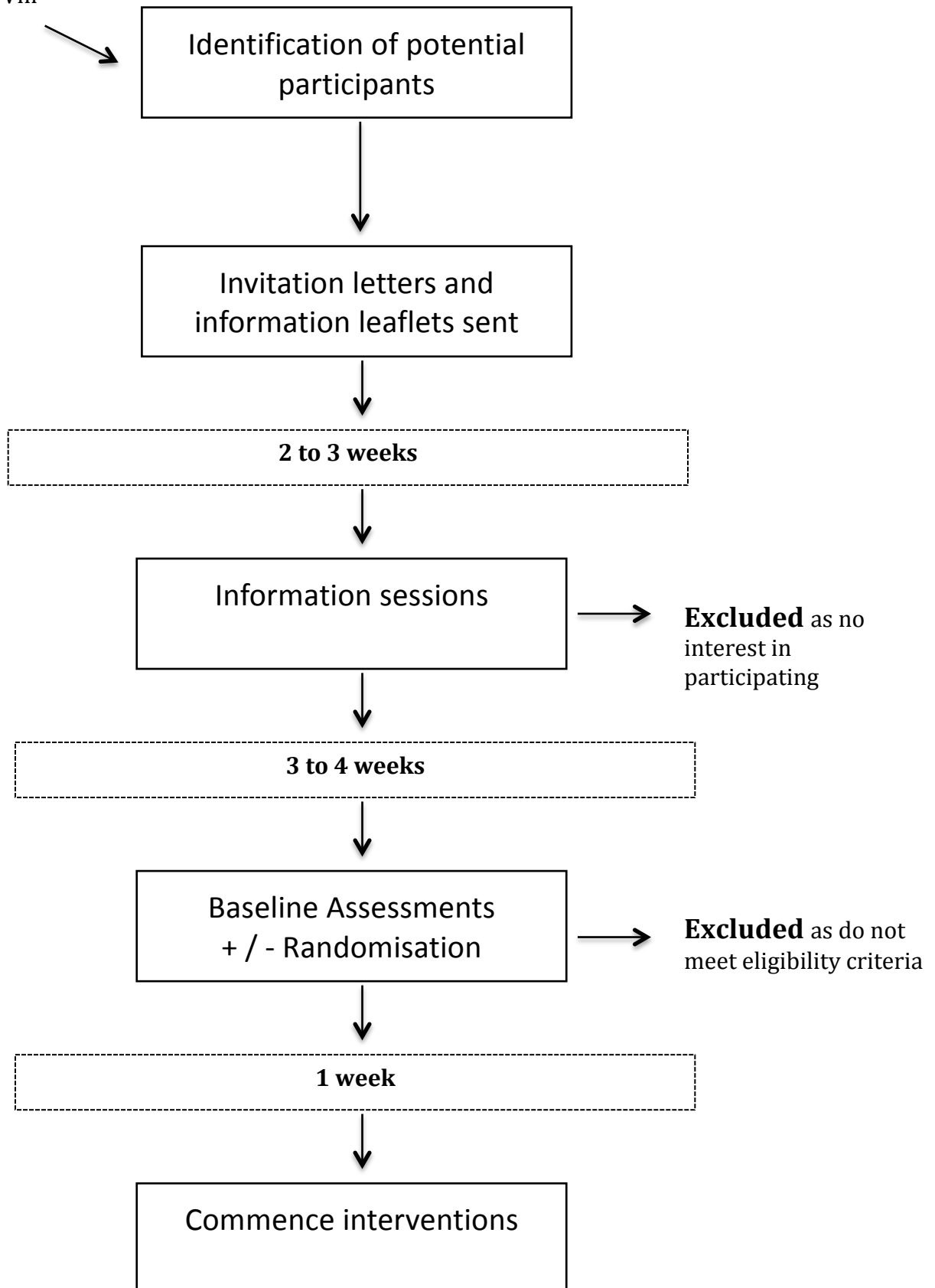

Supplement: Additional file 2: — Participant flow through the recruitment process. (PDF 170 kb) [file 13063_2015_1042_MOESM2_ESM.pdf]
